# COMPARATIVE EFFECTIVENESS OF CANINE- OR EQUINE-ASSISTED THERAPY FOR RESIDENTS WITH DEMENTIA

**DOI:** 10.1093/geroni/igz038.440

**Published:** 2019-11-08

**Authors:** Martha E Kemeny, Deborah Hutchins, Courtney Gramlich

**Affiliations:** 1 Slippery Rock University, Slippery Rock, Pennsylvania, United States; 2 Slippery Rock University Storm Harbor Equestrian Center, Slippery Rock, Pennsylvania, United States

## Abstract

Older adults with dementia may manifest symptoms such as apathy, withdrawal, and aggressive actions (NIA, 2019). Even surrounded by people in LTC, residents with dementia may lack social engagement. Non-pharmacologic approaches are promising (Brodaty & Arasaratnam, 2012) and are reflected in the newer CMS F-tag guidelines (CMS, 2017), but there remains a gap in research about the most effective approaches for LTC residents. Limited research exists on animal-assisted interventions (AAI) as a non-pharmacological approach to increase engagement in people with dementia (Friedman, Thomas & Chung, 2015; Huff-Mercer, 2015) and no known research compares equine- and canine-assisted therapy protocols. This study employed a within-subject alternating-treatments design for three single subjects. Completing the similar tasks of grooming, walking, and interaction, the independent variables are two conditions: 1) Condition A: Equine-assisted activity 2) Condition B: Canine-assisted activity. Using the Dementia Interview Rating (Strauss & Sperry, 2002), apathy was measured before and after each phase. During each session, three outcomes were measured: 1) Engagement in Preferred Activities Scale (Nelson et al., 2014) was used to measure the level of engagement in the interaction; 2) Heart rate variability, a measure of coherence, was measured using the Emwave pro (Heart Math Institute, 2018), and 3) targeted observed social responsiveness (initiation, verbal response, non-verbal response, motor response to one-step instructions) using a smart tablet application. Preliminary results suggest past experience/preference impact the individual client’s response to differing protocols. Effective non-pharmacological interventions for older adults with dementia are an essential alternative to current practice.

